# Proanthocyanidins as a Potential Novel Way for the Treatment of Hemangioma

**DOI:** 10.1155/2021/5695378

**Published:** 2021-01-02

**Authors:** Ran Tang, Dehai Xian, Jixiang Xu, Huiling Peng, Shihong Pan, Jianqiao Zhong

**Affiliations:** ^1^Department of Dermatology, The Affiliated Hospital of Southwest Medical University, Luzhou 646000, China; ^2^Department of Anatomy, Southwest Medical University, Luzhou 646000, China

## Abstract

Hemangioma, the most common benign vascular tumor, not only affects the appearance and psychology but also has a life-threatening potential. It is considered that clonal vascular endothelial cell proliferation and excessive angiogenesis are responsible for hemangioma pathogenesis, in which abnormal cytokines/pathways are closely implicated, primarily including high expression of hypoxia-inducible factor-1*α* (HIF-1*α*) and vascular endothelial growth factor (VEGF) as well as their downstream pathways, especially phosphatidylinositol 3-kinase (PI3K)/protein kinase B (Akt). These further stimulate the migration and proliferation of vascular endothelial cells and promote the formation of new vessels, ultimately leading to the occurrence and development of hemangioma. Proanthocyanidins are naturally active substance from plants and fruits. They possess multiple functions like antiproliferation, antiangiogenesis, and antitumor. It has been demonstrated that proanthocyanidins effectively work in various diseases via inhibiting the expression of various factors, e.g., HIF-1*α*, VEGF, PI3K, and Akt. Considering the pathogenesis of hemangioma and the effect of proanthocyanidins, we hold a hypothesis that proanthocyanidins would be applied in hemangioma via downregulating cytokine/pathway expression, suppressing vascular cell proliferation and arrest abnormal angiogenesis. Taken together, proanthocyanidins may be a potential novel way for the treatment of hemangioma.

## 1. Introduction

Hemangioma, a common benign vascular tumor, is closely associated with excessive angiogenesis/vasculogenesis that early emerges from childhood. It not only occurs on the skin but also involves other organs [1, 2]. Although hemangioma is a benign tumor, it seriously affects the appearance and psychology, even threatens life when hemangioma appears in some specific-functional organs, e.g., visual impairment, respiratory congestion, hepatic hemangioma bleeding, and congestion heart failure [3, 4]. Generally, hemangioma grows rapidly in infancy [5]; subsequently, part spontaneous regression emerges from one year to six years or more [6, 7]; nevertheless, some hemangiomas rarely subside but leave residuals, such as telangiectasias, fibrofatty, scars, and pigmentation [8]. Although the exact etiology of hemangioma still keeps unclear, emerging evidence indicates that hypoxia-induced vascular endothelial cell proliferation and abnormal angiogenesis are mainly involved in hemangioma. Moreover, several reports have implied that hypoxia-inducible factor-1*α* (HIF-1*α*) and vascular endothelial growth factor (VEGF), highly expressing in hemangioma, are major two contributors to vascular endothelial cell proliferation and abnormal angiogenesis [9–11]. Currently, a variety of therapies have been applied in hemangioma including systemic/topical drugs (e.g., corticosteroids, immunosuppressant, propranolol, and itraconazole), pulsed dye laser treatment, and surgery [12]. As a first-line treatment for hemangioma, propranolol is usually used in hemangioma. A clinical trial showed that propranolol exhibited a 100% of improvement rate in fast halt of hemangioma proliferation and a 87% of that in hemangioma regression after 31 patients with rapidly proliferating infantile hemangioma (IH), who had functional or cosmetic defects, were given with propranolol at a dose of 3 mg/kg/day [13]; undoubtedly, propranolol is effective, but some side effects, like *bronchospasm*, *bradycardia*, *hypotension*, *hyperkalemia*, *hypoglycemia*, *etc*., frequently prevent its application in hemangioma [14–17]. Another classical therapy, corticosteroid also displays an optimistic effect on hemangioma; for example, daily systemic administration of prednisone at 2-3 mg/kg seemed to be available for hemangiomas of infancy, accompanied with a 84% of effective rate and a 36% of rebound rate [18]; nevertheless, this vehicle often stands still due to its numerous adverse reactions, e.g., *reversible Cushing*'*s phase*, *mood disorders*, *gastric irritation*, *symptoms of weight gain*, *and adrenal suppression* [19–21]. Recently, laser therapy is more popular in infantile hemangioma. It was reported that regardless of short or long pulse laser treatment was quite effective in infantile hemangiomas [22]; however, traumatic impairments and high cost stop laser therapy. Consequently, these vehicles are limited to application owing to their unbearable side effects or high expenditure. The recently clinical trials about hemangioma treatment are shown in Table 1.

Recently, numerous studies have demonstrated that natural extracts, proanthocyanidins in particular, possess potent antiangiogenic, antiproliferative, immuno-suppressive, anti-inflammatory, and antineoplastic activities, accompanied with few side effects and even application in infants and pregnant women [28, 29]. Proanthocyanidins are abundant in plants and fruits, especially in grape seeds, red wine, cranberries, glyptostroboides, and metasequoia. A serial of evidence supports that proanthocyanidins provide safe and effective ways for many disorders, such as cardiovascular disease, osteoarthritis, diabetes, and oral cancer, primarily by suppressing cell proliferation and preventing angiogenesis/vasculogenesis [30–32]. Nevertheless, rare studies focus on the role of proanthocyanidins in control of hemangioma. Given that hemangioma is an angiogenesis-related vascular tumor and proanthocyanidins have the capabilities of antiangiogenesis, antiproliferation, and antitumor, we put forward the possibility of proanthocyanidins in controlling hemangioma. Therefore, the potential evidence of proanthocyanidins for treating hemangioma is reviewed.

## 2. Pathogenesis of Hemangioma

Although the etiology of hemangioma remains unknown, it is thought that hemangioma is mostly derived from uncontrolled angiogenesis that is generated by the clonal proliferation of vascular endothelial cells [9]. In this process, hypoxia plays a crucial role. It potently promotes vascular proliferation and induces the transcription factor HIF-1 production, which in turn aggravates angiogenesis. As the pivotal factor of hypoxia, HIF-1 (comprising HIF-1*α* and HIF-1*β*) is crucial to the regulation of hypoxia-induced genes [33]. It is stimulated by hypoxia via several mechanisms involving stabilization of HIF-1 protein and various signaling pathways, such as VEGF, phosphatidylinositol-3-kinase (PI3K)/protein kinase B (Akt), p70 ribosomal protein S6 kinase (p70S6K), and mechanistic target of rapamycin (mTOR) [34–38]. Numerous reports have confirmed that there is a significant increase in the protein and mRNA expression of HIF-1 in hemangioma compared with normal vascular tissue [39, 40].

As a potent proangiogenic molecule, HIF-1*α* actively takes part in the formation of new vessels and the proliferation of endothelial cells in hemangioma [41]. The expression and activity of HIF-1*α* is regulated by intracellular oxygen concentration [42]. Under physiological conditions, HIF-1*α* protein is rapidly degraded at the sufficient oxygen level. Under hypoxia conditions, however, HIF-1*α* protein is deposited in the nucleus and forms an active complex with HIF-1*β*, which further activates the transcription of HIF-1*α* target genes [43]. HIF-1*α* level is directly proportional to the activity and severity of hemangioma, and its overexpression is closely implicated in the increased vascularity, invasion, and progression of tumor [44, 45]. Chang et al. discovered that there was a higher level of HIF-1*α* in the proliferative phase of hemangioma than that in the regress phase; but a decrease of HIF-1*α* emerged from the serum of hemangioma patients after systemic sclerotherapy [46]. More importantly, HIF-1*α* could induce the expression of a series of genes, VEGF gene in particular, then stimulate the proliferation and migration of vascular endothelial cells, ultimately lead to the formation of new vessels [47, 48].

VEGF, the downstream of HIF-1*α*, is a vital signaling protein that stimulates angiogenesis and vasculogenesis [49]. It plays a critical role in promoting new blood vessel formation during embryonic growth and tissue repair [50, 51]. As one of the most powerful angiogenic factor in angiogenesis, VEGF effectively works in enhancing the proliferation and permeability of vessels, encouraging the migration of vascular endothelial cell, inhibiting the apoptosis of vascular endothelial cells and facilitating the formation of new vessels [52, 53]. By binding to receptor tyrosine kinases (RTKs), VEGF positively regulates the activity of the vascular endothelial growth factor receptors (VEGFR) [54] and further mediates mitogenic signals via activating both VEGFR1 and VEGFR2 [2]. VEGFR1 mainly expresses on the surface of monocytes and macrophages, VEGFR2 predominating on that of vascular endothelial cells and embryonic precursor cells [55, 56]. In normal endothelial cells, VEGFR-1 is similar to the decoy receptor of VEGF, which binds to VEGF to prevent the activation of VEGF/VEGFR-2; in hemangioma endothelial cells, however, the activity of VEGFR-1 and *β*1 integrin markedly decreases, and VEGF level significantly increases, thereby facilitating VEGFR-2 activation [57]. Despite less affinity for VEGF, VEGFR2 has a higher signal transduction activity than VEGFR1 and thus mediates cell leakage and vascular permeability in response to VEGF [58–60]. VEGFR-1 indeed favors to combine with VEGF, but it scarcely transmits the signal after binding. Instead, the combination of VEGFR-2 and VEGF tends to trigger the specific signals to stimulate endothelial cell proliferation and induce angiogenesis.

**Figure 1:**
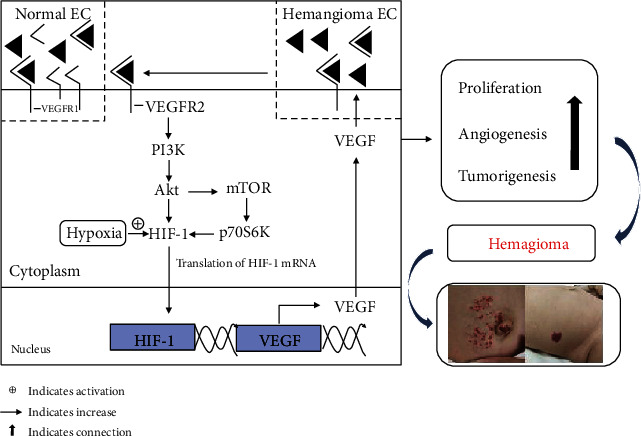
The possible pathogenesis of hemangioma.

It is therefore considered that VEGF/VEGFR2 signaling is a crucial signal transducer in both physiologic and pathologic angiogenesis. Jinnin et al. showed that VEGFR1 reduction in hemangioma facilitated VEGF-dependent VEGFR2 signal activation, further stimulating the downstream pathways (primarily PI3K/Akt signaling pathway) and contributing to the proliferation of vascular endothelial cells and the formation of new vessels [57, 61]. The PI3K/Akt pathway widely expresses in cells to regulate cell survival and proliferation, which is activated by the VEGF-dependent VEGFR2 signal enhancement in hemangioma endothelial cells [62–64]. Akt (also called serine/threonine protein kinase B) is recognized by PI3K that produces 3-phosphoinositide lipids to cause activation of downstream signals; both together play a dominant role in a serial of biological process, e.g., gene expression, cell transformation, cell cycle, cell survival, and vascular trafficking [65, 66]. However, abnormal activation of PI3K/Akt signaling pathway significantly inhibits the spontaneous apoptosis of hemangioma endothelial cells during hypoxia through modulating the downstream components mTOR and p70S6K [67, 68]. On the other side, mTOR also promotes HIF-1 translation and VEGF expression at the appearance of phosphorylated p70S6K [69, 70]. Anyhow, HIF-1 is initially activated, and its downstream pathway VEGF is in turn stimulated in the presence of hypoxia, then activating PI3K/Akt signaling pathway, finally promoting vascular proliferation and resulting in hemangioma occurrence via the regulation of mTOR and p70S6K, which forms an autocrine loop of VEGF signaling via activation of VEGFR2. The pathogenic mechanism of hemangioma is summarized in Figure 1.

The decreased VEGFR1 expression in hemangioma endothelial cells facilitates VEGFR2 persistent activation, which contributes to the combination of VEGFR-2 with VEGF. HIF-1, meanwhile, is activated in the presence of hypoxia, further exciting its downstream VEGF; then, the activated VEGF combines with its receptor VEGFR2, which in turn activates the downstream pathway PI3K/Akt. PI3K and its downstream Akt, on the one hand, directly provoke HIF-1; on the other, they activate their downstream target mTOR/p70S6K to indirectly promote HIF-1 translation, collectively encouraging VEGF activation. As a result, it forms an autocrine loop of VEGF signaling. Above changes ultimately enhance vascular endothelial cell proliferation, accelerate angiogenesis, and trigger hemangioma formation.

## 3. Characteristics and Applications of Proanthocyanidins

Proanthocyanidins, a kind of natural substance, are derived from fruits and plants such as grape, apple, and black currant [71]. They belong to oligo- or/polymeric end products of flavonoidsin, in ingredient containing catechin, epicatechin, gallic acid, or epigallocatechin subunit chains doubly linked by C4-C6 and C4-C8 interferon bonds [72, 73]. As the most classical form of proanthocyanidins, procyanidins are mainly condensed from catechin or epicatechin, which may present as dimer, trimer, tetramer, and decamer in different conditions [74]. Many isomers exist in dimer due to the different conformation or bonding position of the two monomers. At present, eight structural forms are identified, respectively, called Bl-B8; among them, B1-B4 is formed by C4-C8 bond, whereas C4-C6 bond constitutes B5-B8 [75].

Proanthocyanidins are commonly absorbed as monomers that are widely bound in the liver and then released, circulating in the body or accumulating in tissues before excretion from the kidney, while some of them return to the intestine through bile [76, 77]. Besides, some dimers and trimers may be absorbed in the small intestine [78], but most of them will be depolymerized and absorbed as monomers or metabolized by intestinal flora before passing through the colon [79]. Proanthocyanidins possess many activities, e.g., antiangiogenesis, antiproliferation, antioxidation, anti-inflammation, and antitumor [28, 29]. Owing to their powerful effects of antiangiogenesis and antiproliferation, proanthocyanidins have a wild range of application in various angiogenesis-related diseases. Numerous studies have demonstrated that proanthocyanidins could prevent angiogenesis-related damage via mediating in a serial of signaling pathways [80–84]. Angela et al. found that proanthocyanidins availably mitigated the symptoms of osteoarthritis (OA), an angiogenesis-related disorder in osteoarticulation, through suppressing VEGF signaling pathway [85]. Increasing evidence has confirmed that proanthocyanidins are the powerful inhibitor of VEGF and thereby quite excellent in controlling angiogenesis in vitro and in vivo by decreasing VEGF expression, preventing endothelial cell migration and inhibiting vascularization [81, 86–89]. They could directly bind to VEGF molecule and competitively prevent VEGF coupling with its receptor VEGFR-2 through interacting with intracellular components which is involved in VEGFR-2 phosphorylation [90]. Just as above proanthocyanidins alleviated OA, they worked at least in part through targeting VEGF and its receptor; therefore, proanthocyanidins would be a profoundly promising approach to OA treatment by targeting VEGF. Apart from that, proanthocyanidins potently function in another major group of angiogenesis-related diseases, namely, tumor disorders. Attributing to their antiangiogenic and antitumor activities, proanthocyanidins may be a potential antitumor agent under various signaling pathways to participate in cell survival, death, migration, and invasion [91]. It was shown *in vitro and in vivo* studies that proanthocyanidins inhibited esophageal adenocarcinoma via inactivation of PI3K/Akt/mTOR signal [92]. Meanwhile, Zhang et al. demonstrated that proanthocyanidins had a strong growth inhibition on cisplatin-resistant cells through suppressing angiogenesis and promoting G1 cell cycle arrest; they also decreased the expression of VEGF and HIF-1*α* by targeting Akt/mTOR/p70S6K/4E-BP-1 pathway and thereby inhibited angiogenesis [80]. Moreover, proanthocyanidins could inhibit the hypoxia-simulated tumor angiogenesis and cell invasion in a HIF-1*α*-dependent manner [93]. For example, hypoxia could activate HIF-1*α* and matrix metalloproteinases (MMPs) to stimulate the invasion and migration of tumor cell; however, the mRNA and protein levels of hypoxia-induced MMPs significantly decreased in the presence of proanthocyanidins [94]. Collectively, proanthocyanidins effectively inhibit HIF-1*α* expression by blocking the activation of PI3K/Akt/mTOR pathways [94]. Above findings indicate that it is possible for proanthocyanidins, as a potential antiangiogenesis candidate, to prevent hemangioma angiogenesis through targeting the HIF-1/VEGF signals via inactivating PI3K/Akt pathway along with their downstream components mTOR/p70S6K. In addition, numerous clinical trials of proanthocyanidins have been performed for the treatment of various diseases in patients, in healthy subjects, in infants, and even in pregnant women [29, 95, 96]. These studies consistently show that proanthocyanidins are safe and effective, proving their real application in diverse diseases. However, the application of proanthocyanidins in hemangioma has not yet been reported until now.

Table 2 summarizes the abovementioned characteristics of proanthocyanidins, including chemical structure, distribution, functions, metabolism, applications, and adverse reactions.

### 3.1. Hypothesis for Proanthocyanidins in the Management of Hemangioma

**Figure 2:**
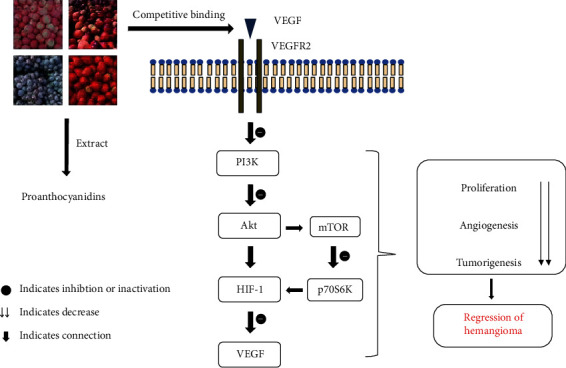
The hypothetical process of proanthocyanidins in management of hemangioma.

Given that abnormal angiogenesis and vascular endothelial cell proliferation are primarily involved in hemangioma and proanthocyanidins have the properties of antiangiogenesis and antiproliferation, it is postulated that proanthocyanidins may be greatly available in management of hemangioma. Here, the detailed imaginary idea is formulated as following: (1) hemangioma is a common benign vascular tumor that mainly involves uncontrolled angiogenesis and abnormal vascular endothelial cell proliferation [9]. (2) Hypoxia-induced HIF-1*α* and VEGF are central contributors to the pathogenesis of hemangioma [33–35]. (3) HIF-1*α* is initially induced by hypoxia and then activates VEGF, further to promote vascular proliferation and aggravates angiogenesis under a serial of signals, e.g., PI3K/Akt, mTOR, and p70S6K kinase [33–38]. (4) The imbalance of angiogenic/antiangiogenic factors and the increase of HIF-1*α* or/and VEGF have been found in hemangioma lesion [48, 49]. HIF-1*α* can activate the proangiogenic factor VEGF, whereas proanthocyanidins facilitate HIF-1*α* levels decrease [81, 88–90]. (5) Proanthocyanidins are natural active substances with antiangiogenic, antiproliferative, and antitumor activities nearly without few adverse reactions [28, 29]. (6) Proanthocyanidins effectively prevent cell proliferation, angiogenesis, and tumorigenesis via suppressing VEGF and HIF-1*α* activation [82–85]. (7) Proanthocyanidins binding to VEGF inhibit HIF-1*α* translation and the decrease VEGF expression through mediating the PI3K/Akt and mTOR/p70S6K signaling pathways, thereby arresting the proliferation, invasion, and migration of tumor cell [88, 90]. The hypothetical process of proanthocyanidin control hemangioma is shown in Figure 2.

Proanthocyanidins are initially extracted from numerous natural fruits and plants, e.g., grapes, apples, and blackcurrants. They, then, competitively combine with VEGF to prevent HIF-1*α* activation via directly stopping PI3K/Akt pathway or indirectly blocking mTOR/p70S6K signal, further downregulating the VEGF expression. As a result, significant reductions emerge from the proliferation, invasion, and migration of tumor cell. Ultimately, hemangioma is possible to be controlled effectively.

### 3.2. Clinical Significance

Hemangioma has long been a hotspot in the field of dermatology research. Although various medications and physiotherapies are used to treat hemangioma, these vehicles are constricted by serious adverse reactions, high cost, and traumatic influence. Proanthocyanidins, a kind of natural plant extracts with few side effects, are expected to be applied in hemangioma. Owing to their antiangiogenic and antiproliferative activities, proanthocyanidins will likely decrease the levels of PI3K, Akt, HIF-1*α*, and VEGF and inhibit vascular endothelial cell proliferation, angiogenesis, and tumorigenesis. Last but not least, proanthocyanidins are safe for infants, pregnant woman, and the elderly [29, 95, 96].

### 3.3. Future Research

Herein, we speculate that proanthocyanidins have powerful efficacy on hemangioma. To prove the above hypothesis and clarify the effects of proanthocyanidins on hemangioma, further studies are warranted *in vitro and in vivo*. Firstly, animal experiments will be performed in well-established hemangioma mice models to investigate the effectiveness of dietary proanthocyanidins. Subsequently, the therapeutic mechanism of proanthocyanidins on hemangioma should be explored in a cell model and three-dimensional model. Meanwhile, the related parameters are determined through a serial of molecular biotechnologies. Finally, randomized controlled clinical trials may provide a scientific basis for more research and clinical applications of proanthocyanidins.

## 4. Conclusion

Above all, hemangioma is a benign vascular tumor, frequently occurring in children, which has a negative impact on quality of life even threatens life. Although the specific pathogenesis of hemangioma has not been fully elucidated, it is currently regarded as multifactorial condition involving vascular endothelial cell proliferation, uncontrolled angiogenesis, and abnormal cytokines/pathways under hypoxia induction, e.g., HIF-1*α*, VEGF, and PI3K/Akt. By targeting these crucial points, hemangioma is hopeful to be cured safely and effectively. At present, drug treatments for hemangiomas include propranolol, glucocorticoid, interferon, and others. These vehicles indeed exhibit improved effect on hemangioma, but the cost and side effects stop patients from adopting them. Consequently, cost-effective and safe treatments are required for hemangioma. Here, we expound the pathogenesis of hemangioma and enumerate the clinical application of proanthocyanidins. It indicates that proanthocyanidins would be an ideal alternative for the control of hemangioma. Indeed, we will carry out *in vitro and in vivo* experiments to validate the proposed signaling pathways in hemangioma and confirm the improvements in hemangioma parameters after proanthocyanidin treatment.

## Figures and Tables

**Table 1:** Clinical trials of various treatments in hemangioma.

Author, year	Intervention	Cases (F/M)	Age (Mon)	Dose	Location	Efficiency	Side effects
Holmes et al. 2011 [13]	Propranolol	31	1.2-9.7	3 mg/kg/d	81% facial 10% upper limbs 6% perineum 3% lower limbs	87%	^∗^1 hypotension
							2 gastro-esophageal
							1 bronchiolitis
Li et al. 2015 [23]	Propranolol	17 (10/7)	2-11	1 mg/kg/d	100% facial	—	2 diarrhea
							1 sleep disturbance
Léaute-Labrèze et al. 2015 [24]	Propranolol	456 (325/131)	1–5	1-3 mg/kg/d	70% facial 30% nonfacial	60%	6 hypotension
							3 bronchospasm
							2 bradycardia
							2 hypoglycemia
							Other risks (diarrhea, sleep disorder, bronchitis, etc.)
Hogeling et al. 2011 [25]	Propranolol	19 (14/5)	2-60	1-2 mg/kg/d	90% facial 5% torso 5% limb	97%	1 transient cool extremities
							4 bronchiolitis
							2 hemangioma ulceration
							4 disturbances
Kim et al. 2017 [26]	Corticosteroid	47 (39/8)	0.8-8.0	2 mg/kg/d	76% facial 24% nonfacial	91.94%	1 hypotension
							7 hypotension
							2 growth disability
Tay et al. 2012 [22]	Pulsed dye laser	23 (19/4)	2.5-19	595 nm	91% facial 9% nonfacial	100%	3 hyperpigmentation
							4 hypopigmentation
							3 mild textural changes
Kono et al. 2011 [27]	Pulsed dye laser	A: 26(16/10) B: 26(14/12)	1-3	A: 585 nm B: 595 nm	100% facial	A: 88% B: 100%	11 hypopigmentation
							6 hyperpigmentation
							7 texture change
							1 ulcer formation

^∗^ indicates the number of cases with side effects.

**Table 2:** Structure, distribution, and characteristics of proanthocyanidins.

Structure	Distributions	Functions	Metabolism	Application	Side effects
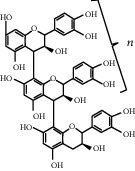	Fruit/seeds/peel (grapes, apples, blackcurrants, etc.)	Antiangiogenesis	Broken down into monomers in the intestine	Cardiovascular disease	No obvious side effects, even in pregnant women
*n* = 2‐−4 oligomeric proanthocyanidins	Leaves	Antiproliferation	Widely bound in the liver	Neurodegenerative disorder	
*n* ≥ 5 polymeric proanthocyanidins	Flower	Antioxidation	Excreted by the kidneys	Metabolic disease	
	Root/stem	Anti-inflammation		Osteoarthritis	
		Antitumor		Cancer	

## Supplementary Materials

Supplementary Materials Graphical abstract: proanthocyanidins have the potential to treat hemangioma. HIF-1*α*: hypoxia-inducible factor-1*α*; VEGF: vascular endothelial growth factor; VECs: vascular endothelial cell; ⊖ indicates “inhibit or suppress”; ↓ indicates decrease.
