# Supplementary material for: Proanthocyanidins as a Potential Novel Way for the Treatment of Hemangioma
Source: Biomed Res Int. 2021 Jan 2;2021:5695378. doi: 10.1155/2021/5695378 (PMC7801061; doi:10.1155/2021/5695378)
Supplement: Supplementary Materials — Graphical abstract: proanthocyanidins have the potential to treat hemangioma. HIF-1α: hypoxia-inducible factor-1α; VEGF: vascular endothelial growth factor; VECs: vascular endothelial cell; ⊖ indicates “inhibit or suppress”; ↓ indicates decrease. [file 5695378.f1.docx]

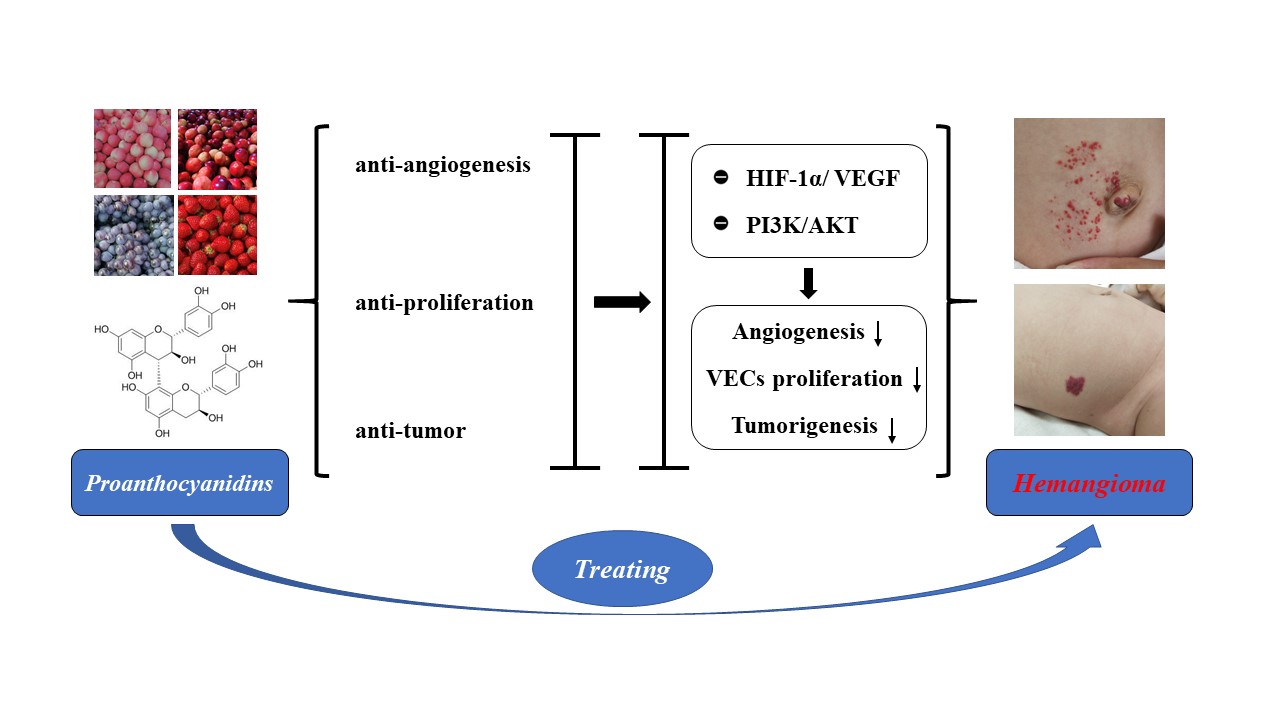


Graphical Abstract: Proanthocyanidins have the potential to treat hemangioma. HIF-1a, hypoxia-inducible factor-1a; VEGF, vascular endothelial growth factor; VECs, vascular endothelial cell;
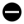
 indicates “inhibit or suppress”; ↓ indicates decrease.
